# Maternal Characteristics of Women Exposed to Hypnotic Benzodiazepine Receptor Agonist during Pregnancy

**DOI:** 10.1155/2014/945621

**Published:** 2014-04-10

**Authors:** Bjarke Askaa, Espen Jimenez-Solem, Henrik Enghusen Poulsen, Jon Traerup Andersen

**Affiliations:** ^1^Department of Clinical Pharmacology, Bispebjerg Hospital, Bispebjerg Bakke 23, 2400 Copenhagen, Denmark; ^2^Laboratory of Clinical Pharmacology Q7642, Rigshospitalet, 2100 Copenhagen, Denmark

## Abstract

*Background*. There is little knowledge regarding the characteristics of women treated with hypnotic benzodiazepine receptor agonists (HBRAs) during pregnancy. In this large Danish cohort study, we characterize women exposed to HBRA during pregnancy. We determined changes in prevalence of HBRA use from 1997 to 2010 and exposure to HBRAs in relation to pregnancy. *Methods*. We performed a retrospective cohort study including 911,017 pregnant women in the period from 1997 to 2010. Information was retrieved from The Danish Birth Registry and The Registry of Medicinal Product Statistics to identify pregnant women redeeming a prescription of HBRAs. *Results*. We identified 2,552 women exposed to HBRAs during pregnancy, increasing from 0.18% in 1997 to 0.23% in 2010. Compared to unexposed women, exposed women were characterized by being older, with higher BMI, in their third or fourth parity, of lower income and education level, more frequently smokers, and more likely to be comedicated with antipsychotic, anxiolytic, or antidepressant drugs (*P* < 0.0001). *Conclusion*. Women using HBRAs during their pregnancy differ from unexposed women in socioeconomic factors and were more likely to receive comedication. The consumption of HBRAs was reduced during pregnancy compared to before conception.

## 1. Objective

There is an uncertainty regarding the pharmacological treatment of sleeping disorders during pregnancy due to the fear of negative birth outcomes. It has been reported that 64–88% of pregnant women in western countries experience disturbed sleep during pregnancy, in contrast to 20–38% of women in the general population [1, 2].

The preferred medical treatment for sleeping disorders during pregnancy is hypnotic benzodiazepine receptor agonists (HBRAs), because of a decreased abuse and addictive potential compared to benzodiazepines [3, 4]. In Denmark, zopiclone, zolpidem, and zaleplon are the only used HBRAs. They bind to the benzodiazepine receptor subunit of the GABA receptors and peak plasma concentrations are attained 1-2 hours after dosing [3]. HBRAs are used in the treatment of insomnia and metabolized primarily in the liver with a relatively short elimination half-life of 1–5 hours [5, 6]. Zolpidem crosses the human placenta and is detectable in the umbilical cord but is rapidly cleared from the fetus' circulation [7]. There are no reports showing an increased risk of malformations for infants conceived of women using HBRAs [7–12].

The purpose of this retrospective study is to determine the prevalence of HBRAs use and characteristics among pregnant women from 1997 to 2010.

## 2. Study Design

All births in Denmark between 1 January 1997 and 31 December 2010 (*n* = 918,041) were identified. 6,472 pregnancies were excluded from the study due to missing information in gestational length or if gestation length was recorded as less than 155 days or more than 315 days. The final cohort consisted of 911,569 births. The Medical Birth Registry [13] consists of individual-level data on the mother and father, including a unique identification number, birth weight and length, sex of offspring, parity, gestational age of the offspring, pregestational body mass index (BMI) of the mother, and information on smoking during pregnancy. More than 99.5% of births in Denmark since 1978 are registered in the Medical Birth Registry [14]. Information on prescription medication use was collected from the Register of Medicinal Product Statistics (the National Prescription Register) [15, 16]. The register contains individual-level data on all prescribed drugs dispensed at all pharmacies in Denmark since 1995. Completeness has previously been estimated to be 97.5% [17]. Information on household income and educational length was from Statistics Denmark which holds information from Danish education registers and registers on personal income and transfer payments [18, 19].

Exposure was defined as redemption of a prescription of zopiclone (Anatomical Therapeutic Chemical Classification (ATC) N05CF01), zolpidem (ATC N05CF02), or zaleplon (ATC N05CF03) during pregnancy. Exposure to antipsychotics (ATC N05A), anxiolytics (ATC N05B), and selective serotonin reuptake inhibitors (SSRIs) (ATC N06A) was defined as redemption of at least one prescription during pregnancy.

Differences in baseline characteristics between exposed and unexposed were analyzed using a logistic regression model with the following variables included: maternal age (five categories: <20, 20–24, 25–29, 30–34, and ≥35 years), income (categorized as quartiles), length of education (<144, 144–155, 156–179, and >179 months), number of offspring in the current pregnancy (1, 2, and ≥3), parity (1, 2, 3, and ≥4), smoking during pregnancy (yes/no), use of antipsychotics during the pregnancy (yes/no), use of anxiolytics during the pregnancy (yes/no), use of SSRIs during the pregnancy (yes/no), and year of delivery (as a continuous variable). Since information on BMI was only available from 2004, a separate logistic regression model was constructed including the above mentioned variables and BMI (five categories: <18.5, 18.5–24.9, 25.0–29.9, 30.0–34.9, and ≥35).

Data on maternal age, parity, number of offspring, education, and income had less than 1% missing values. Data on smoking status was missing in 3.2% of the records and information on education was missing in 3.1% of the records. BMI was only available from 2004 and missing in 6.5% of the records.

For all analyses, a two-sided value of *P* < 0.05 was considered to be statistically significant.

In Denmark, the Act on Processing of Personal Data does not require ethical permission or obtained written informed consent for anonymized retrospective register studies. All data were held by Statistics Denmark and were only made available with encrypted personal information. This ensured that no individuals could be identified. The Danish Data Protection Agency approved the study (number 2008-41-2517).

All data management and analyses were performed using SAS software, version 9.2 (SAS Institute Inc., Cary, NC, USA). We report our findings according to strengthening the reporting of observational studies in epidemiology (STROBE) [20].

## 3. Results

In the period from 1997 to 2010, we surveyed 911,017 pregnancies ending in a live birth in Denmark, of which 2,552 (0.3%) were exposed to an HBRA at some point during pregnancy.

### 3.1. Maternal Characteristics

Pregnant women exposed to HBRAs were more likely to have higher parity (*P* < 0.001), have a shorter education (*P* < 0.001), have lower household income (*P* < 0.001), be older (*P* < 0.001), have higher BMI (*P* < 0.001), and be more frequently smoking during pregnancy (*P* < 0.001) compared with unexposed women (Table 1).

### 3.2. Exposure in relation to Pregnancy

In our cohort, the consumption of HBRAs declined considerably from 3,443 (0.4%), 12 weeks before conception, to 1,688 (0.2%), in the first trimester of pregnancy. This trend continued to a low point of 571 (0.1%) in the second trimester. The number of exposed women increased during the last trimester of pregnancy and 12 weeks after delivery to 741 (0.1%) and 1,888 (0.2%), respectively (Figure 2). There was a 45% (*P* < 0.001) decrease in the use of HBRAs when comparing the twelve weeks before conception with the twelve weeks postpartum.

### 3.3. Exposure over Time

**Figure 1:**
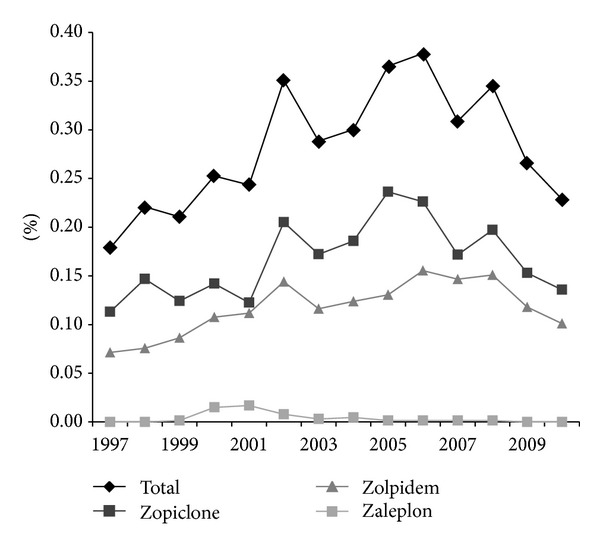
Exposure of hypnotic benzodiazepine receptor agonist (HBRA) among pregnant women from 1997 to 2010. Drug exposure is divided into subgroups of the different HBRAs available in Denmark.

The use of HBRAs among pregnant women increased between 1997 and 2006 from 120 (0.2%) to 246 (0.4%) (*P* < 0.001) users per year and then declined to 145 (0.2%) in 2010 (*P* < 0.001). The choice of HBRAs did not change during pregnancy or the study period. In 2010, zopiclone represented 59% (*n* = 86), zolpidem represented 44% (*n* = 64), and zaleplon represented 0.0% (*n* = 0) of the HBRA use during pregnancy. Zaleplon was mainly used in the period from 2000 to 2002 (Figure 1).

### 3.4. Comedication

Pregnant women exposed to HBRAs, compared with unexposed pregnant women, were significantly more likely to use SSRIs (5.5% versus 1.7%, *P* < 0.001), anxiolytics (15.6% versus 0.5%, *P* < 0.001), or antipsychotics (8.1% versus 0.2%, *P* < 0.001) (Table 2).

## 4. Discussion

With data from the Danish Birth Registry and the Register of Medicinal Product Statistics, we identified all pregnancies in the period from 1997 to 2010 exposed to an HBRA. Among the identified pregnancies, we found 2,552 (0.3%) women exposed to zopiclone, zolpidem, or zaleplon during pregnancy. This exposure rate was higher than in a Swedish study (*n* = 859.455) where only 0.06% of women were exposed to HBRAs during pregnancy, between 1995 and 2004 [21]. Possible explanations for the difference in exposure rates could be different practices in treatment of sleeping disorders or sociocultural differences between the countries.

The results of this study support earlier findings from Sweden [21]. Among pregnant women using HBRAs, we found a higher rate of smokers; the users were older and had shorter education, lower income, and higher BMI compared with pregnant women not treated with HBRAs. Furthermore, higher rate of use of HBRAs was observed after second parity. Overall, the results of the present study may not be generalizable to other patient populations and countries.

### 4.1. Use of HBRAs in relation to Pregnancy

The rate of exposure to HBRAs among pregnant women increased from 1997 to 2006 and then decreased in 2010 to a level 28% higher than in 1997. The 61% decline from 2006 (0.4%) to 2010 (0.2%) has not been described in other studies and is possibly a result of increased focus on potential overuse of benzodiazepines and HBRAs initiated in 2006 by the Institute for Rational Pharmacotherapy in Denmark [22]. In the overall Danish population, the use of benzodiazepines and HBRAs was reduced with 20% and 15%, respectively, between 2006 and 2009 [22]. Earlier studies reported that the use of pharmaceuticals decreases in relation to pregnancy planning or recognition [23, 24]. A similar pattern was seen in our study among pregnant women using HBRAs. This may depend on either patients' or physicians' fear of adverse pregnancy outcomes when exposed to HBRAs during pregnancy or possibly as a natural cause of increased fatigue because of pregnancy.

**Figure 2:**
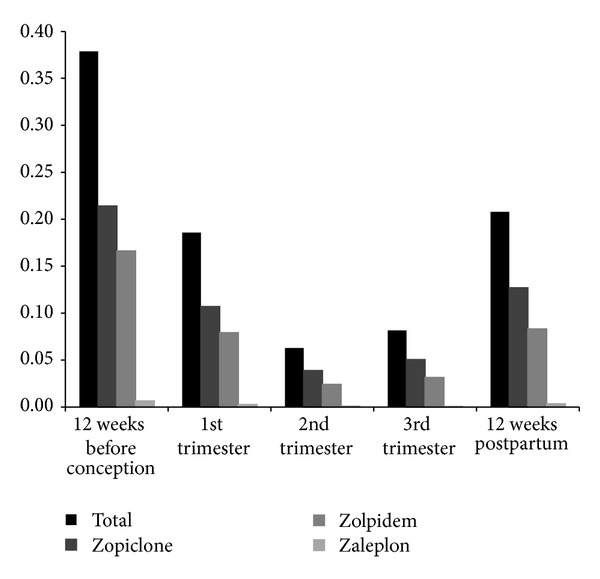
Exposure to hypnotic benzodiazepine receptor agonist during pregnancy (% of all pregnancies). Drugs are divided into subgroups of the different HBRAs available in Denmark.

Previous studies have reported that pregnant women treated with HBRAs are more likely to be comedicated with other psychoactive drugs [21]. These studies report that 25% of women exposed to HBRAs also used antidepressants. When considering that newer antidepressant drugs (SSRIs) are without sedative effect, sleeping disorder can be an ongoing issue for the depressed patient. These depressed patients have been shown to gain improved sleep when comedicated with HBRAs [25, 26]. We accordingly assumed that women consuming HBRAs during pregnancy are more likely to receive SSRIs, antipsychotic, or anxiolytic treatment. Our results show an increased rate of comedication with these drugs (Figure 2).

### 4.2. Strengths and Limitations

In this nationwide cohort study, we included information on all live births in Denmark and their mothers' redeemed prescriptions. We minimized the risk of selection bias by obtaining a complete population regardless of race, education, or income level.

We have no information on the indication or prescribed dosage for HBRA treatment, although in Denmark the only indication for HBRA treatment is sleeping disorders. Furthermore, we did not have information on women discontinuing treatment or women with low adherence, which could lead to misclassification and an overestimation of exposure rates. However, a Dutch study estimated that 94.5% of pregnant women redeeming prescribed medicine were exposed to them [27]. Additionally, the National Prescription Register includes 97.5% of all redeemed prescriptions in Denmark which makes the data representative of the Danish population [17].

One of the main strengths in this study is the completeness of the Danish registers including information on nearly all births and redemptions of prescribed medicine. Furthermore, information contained in the registers is collected prospectively which reduces the risk of recall-bias, given that no information was based on interviews.

## 5. Conclusion

In conclusion, we report that the consumption of HBRAs was reduced from 12 weeks before conception until the third trimester of pregnancy. The consumption of HBRAs by pregnant women in Denmark has increased from 1997 until 2010, although a notable decline over the last three years of the study period was seen. Pregnant women redeeming a prescription for HBRAs were more likely to be older, smokers, and in third or fourth parity and have a lower income, shorter education, and higher BMI compared with unexposed pregnant women. Additionally, we found that exposed women were more frequently in treatment with antipsychotic, anxiolytic, or antidepressant drugs.

## Figures and Tables

**Table 1:** Characteristics of women exposed to hypnotic benzodiazepine receptor agonists (HBRA) during pregnancy.

Characteristic	HBRA exposed pregnant women, *n* = 2552 (%)	Non-HBRA exposed pregnant women, *n* = 909017 (%)	*P* value
Maternal age (years)			<0.001
<20	33 (1.3)	13580 (1.5)	
20–24	231 (9.1)	105215 (11.6)	
25–29	641 (25.1)	307541 (33.8)	
30–34	886 (34.7)	326004 (35.9)	
≥35	761 (29.8)	156677 (17.2)	
Income			<0.001
Lowest quartile	986 (38.6)	227125 (25.0)	
Low quartile	601 (23.6)	227215 (25.0)	
Medium quartile	458 (18.0)	227364 (25.0)	
High quartile	507 (20.0)	227313 (25.0)	
Education (months)			<0.001
<145	946 (37.1)	213097 (23.4)	
145–155	351 (13.8)	141008 (15.5)	
156–179	575 (22.5)	259747 (28.6)	
>179	672 (24.6)	266779 (29.4)	
Offspring			<0.16
1	2429 (95.2)	870635 (95.8)	
2	118 (4.6)	37423 (4.1)	
≥3	5 (0.2)	959 (0.1)	
Parity			<0.001
1	1019 (39.9)	397056 (43.7)	
2	786 (30.8)	332642 (36.6)	
3	457 (17.9)	126631 (14.0)	
≥4	276 (10.8)	46976 (5.2)	
BMI			<0.001
<18.5	69 (2.7)	17993 (2.0)	
18.5–24.9	747 (29.3)	264169 (29.1)	
25–29.9	272 (10.7)	87662 (9.6)	
30.0–34.9	138 (5.4)	32703 (3.6)	
≥35	80 (3.1)	17179 (1.9)	
Smoking			<0.001
Yes	847 (33.2)	162978 (17.9)	
No	1594 (62.5)	716573 (78.8)	

**Table 2:** HBRA exposed and nonexposed pregnant women and combined use of psychoactive drugs.

	HBRA exposed pregnant women, *n* = 2552 (%)	Non-HBRA exposed pregnant women, *n* = 909017 (%)	*P* value
Antipsychotic	206 (8.1)	1593 (0.2)	<0.001
Anxiolytic	373 (14.6)	4763 (0.5)	<0.001
SSRIs	650 (25.5)	14995 (1.7)	<0.001
